# Carbon Nanofibers-Sheathed Graphite Rod Anode and Hydrophobic Cathode for Improved Performance Industrial Wastewater-Driven Microbial Fuel Cells

**DOI:** 10.3390/nano12223961

**Published:** 2022-11-10

**Authors:** Nasser A. M. Barakat, Rasha H. Ali, Hak Yong Kim, Mamdouh M. Nassar, Olfat A. Fadali, Gehan M. K. Tolba, Hager M. Moustafa, Marwa A. Ali

**Affiliations:** 1 Chemical Engineering Department, Faculty of Engineering, Minia University, Minya 61519, Egypt; 2 Department of Nano Convergence Engineering, Jeonbuk National University, Jeonju 54896, Korea; 3 Department of Organic Materials and Fiber Engineering, Jeonbuk National University, Jeonju 54896, Korea

**Keywords:** microbial fuel cells, electrospinning, carbon nanofibers, wastewaters

## Abstract

Carbon nanofiber-decorated graphite rods are introduced as effective and low-cost anodes for industrial wastewater-driven microbial fuel cells. Carbon nanofiber deposition on the surface of the graphite rods could be performed by the electrospinning of polyacrylonitrile/N,N-Dimethylformamide solution using the rod as nanofiber collector, which was calcined under inert atmosphere. The experimental results indicated that at 10 min electrospinning time, the proposed graphite anode demonstrates very good performance compared to the commercial anodes. Typically, the generated power density from sugarcane industry wastewater-driven air cathode microbial fuel cells were 13 ± 0.3, 23 ± 0.7, 43 ± 1.3, and 185 ± 7.4 mW/m^2^ using carbon paper, carbon felt, carbon cloth, and graphite rod coated by 10-min electrospinning time carbon nanofibers anodes, respectively. The distinct performance of the proposed anode came from creating 3D carbon nanofiber layer filled with the biocatalyst. Moreover, to annihilate the internal cell resistance, a membrane-less cell was assembled by utilizing a poly(vinylidene fluoride) electrospun nanofiber layer-coated cathode. This novel strategy inspired a highly hydrophobic layer on the cathode surface, preventing water leakage to avoid utilizing the membrane. However, in both anode and cathode modifications, the electrospinning time should be optimized. The best results were obtained at 5 and 10 min for the cathode and anode, respectively.

## 1. Introduction

Numerous industrial effluents produce large quantities of wastewater with varying compositions and vast hydraulic loads compositions [1,2,3]. Industrial effluents contain a variety of substances, including organic waste, inorganic chemicals, suspended or dissolved particles, and heavy metal effluents [4]. The likelihood of such effluent being uncontrolledly dumped into water bodies will have serious negative effects on the social and natural systems. Because of the great complexity and variety of the contaminants in this wastewater, it must be treated in order to fulfil the necessary criteria for water quality, requiring the use of highly effective and affordable methods [5]. 

The sugar industry is the most prevalent agro-based sector in more than 130 countries, especially in developing ones. This sector today contributes significantly to economic growth and is a key driver of employment creation in many developing countries in Asia, Africa, and South America. The industry is involved in sugarcane processing action to produce raw sugar from more than 70% of the sugarcane produced in the worldwide [6]. The chemicals, feed stocks, and end products utilized in the sugarcane industry affect the quantity and composition of the produced wastewater. While secondary wastewater sources are mostly obtained from barometric condensers, dust removal in the chimney and scrubbers, and cooling of turbines, including washing water, the majority of the wastewater is produced during the cane processing operations, e.g., evaporation, crystallization, and refining [7]. In addition, sugarcane that enters the factory usually contains approximately 70–80% moisture, so 0.7 m^3^ of the wastewater is generated per ton of crushed sugarcane [6,8]. In literature, many studies conducted in different countries reported that about 1 m^3^ of wastewater is produced per ton of cane crushed. Moreover, it was also reported, for around 1.5–2 m^3^ fresh consumed to process 1 ton of cane, 1 m^3^ of wastewater is generated [9]. The discharged wastewater is characterized by high organic loads and intense color. The typical level of BOD_5_ in the generated wastewater is in the range of 50,000–60,000 mg/L while the COD can reach from 110,000 to 190,000 mg/L [10].

In general, for the industrial wastewaters, numerous authors have discussed various wastewater treatment systems in the literature. The kind of pollutants present in wastewater affects the treatment procedures [5]. Some of them are based on advanced oxidation processes [11,12], chemical coagulation [13], bio-coagulation [14], filtration [15], ion exchange [16], aerobic and anaerobic treatment [17], and others [18]. The majority of them need significant financial investments, and their application is constrained mostly due to economic concerns taking precedence over the significance of pollution management.

Paradoxically, it is possible to use the organic pollutants existing in industrial wastewaters as a sustainable and renewable energy source. A wonderful tool that may be used to accomplish this goal is the microbial fuel cell (MFC), which simultaneously treats wastewater and produces electrical energy from it. The MFC is a cutting-edge environmental and energy technology that produces electrical energy from organic contaminants found in wastewater [19,20]. Unfortunately, this promised device has not shifted to the industrial scale yet due to the low performance. Although, many trials have been reported to enhance the generated power from the microbial fuel cells, most of these reports introduced MFC power less than 100 mW/m^2^ even by using special substrates [21]. Consequently, there are numerous trials underway to enhance the performance of the MFC through different ways, such as cathode or anode development [22,23], operating conditions optimization [24], membrane modification [25], and electrolyte adjustment [26]. Moreover, other researchers applied various types of microorganisms [27,28], media (fuel) [27,29], electrode materials/sub materials [30,31], and cell configurations [32,33]. However, anode attracts the most attention among the aforementioned factors affecting the MFC performance due to its strong and direct impact on the generated power and current densities. Consequently, numerous reports have been published about anode modification compared to the other factors [34,35]. 

Nanomaterials can provide marvelous solutions for many hopeless problems. For anode modification, electrospun nanofibers have been exploited to improve the performance. For instance, double layer and Co-doped carbon nanofibers distinctly enhanced the industrial wastewater-based MFC [20,36]. In addition, exo-electrogenic microorganisms, such as the model bacterium *Shewanella oneidensis* or a bacterial consortia found in wastewater, colonized freshly electrospun carbon nanofiber electrodes before being introduced into a lab-scaled system [37]. 

In addition, the carbon nanofiber mats’ nanopores are uncomfortable for the micro-scale biocatalyst (microorganisms). In other words, it is difficult for microorganisms to penetrate the carbon nanofiber anode, which negates the benefit of the nanostructure and abolishes the feature of a high surface area. Other methods have thus been used to provide the electrospun carbon nanofiber-based anodes more characteristics. For instance, a hybrid anode made of carboxylated multiwalled carbon nanotubes and carbon nanofibers was electrospun and utilized to increase the performance of MFCs by increasing cell attachment and lowering anode potential [38]. A composite anode made of TiO_2_ (rutile) and carbon nanofibers was studied in an MFC for the same purpose [18]. However, the generated power from aforementioned anodes-based MFCs was not sufficient.

Actually, the packing density of the carbon nanofiber layer is a key factor. In more detail, at low packing density, the microorganisms can penetrate through the nanofiber layer due to presence of large pores. Microorganism insertion through the carbon nanofiber layer creates an excellent conductive network to transfer the electrons. However, after a specific packing density threshold, the pores will be too small to pass the microorganisms, which results in a strong decrease in the generated power. In this study, the aforementioned hypothesis was experimentally proven by the fabrication of carbon nanofibers-attached graphite to be exploited as anode in a sugarcane industry wastewater-based MFC. The results were very satisfactory as the performance was strongly enhanced at the optimized amount of the attached carbon nanofibers.

## 2. Materials and Methods

### 2.1. Materials

For comparison, carbon cloth (CC) and carbon paper (CP) were purchased from Electro Chem. Inc., USA, and utilized as anodes. For all experiments, Pt-loaded carbon cloth (0.5 mg/cm^2^, Electro Chem. Inc., Woburn, MA, USA) was used as a cathode. Polyacrylonitrile (PAN, Mwt 500,000) and N,N-dimethylformammide (DMF) were utilized to generate carbon nanofiber layer on the surface of 1 cm diameter graphite rod. Wastewater from Abu-Korkas Sugar Company, Minya, Egypt was used as anolyte (anode chamber solution). Many samples were collected from the industrial plant, and the average characterization of the used samples is summarized in Table 1.

### 2.2. Anodes Preparation

As a widely used precursor for carbon nanofibers fabrication using the electrospinning technique, a 10 wt% polyacrylonitrile (PAN)/N,N-Dimethylformamide (DMF) was prepared by stirring at 60 °C for 8 h. Using a commercial graphite rod as a collector, the remaining processing parameters of the electrospinning process were as follow: needle diameter of 0.1 mm, needle-to-collector distance of 17 cm, applied DC potential of 15 KV and the process was kept at ambient temperature. Heating under air atmosphere at 250 °C for 1 h was performed for stabilization of the initial electrospun nanofibers. Later on, the graphitization process was performed at 900 °C under nitrogen atmosphere for 1 h. The heating rate in both cases was fixed at 2.5 deg/min.

### 2.3. Cathodes Modification

Poly(vinylidene fluoride) (PVDF, Mn ~275,000, Aldrich, Burlington, MA, USA) was dissolved in DMF solvent to prepare a final solution containing 20 wt% polymer, this solution was used as an electrospun solution. The electrospinning process was performed at almost similar aforementioned conditions, but 20 KV applied voltage was used. The nanofibers were collected at the Pt-free surface of 3.5 × 3.5 cm^2^ Pt-loaded carbon cloth to form hydrophobic surface. The optimum electrospinning time was noticed at 5 min. The electrospun layer was vacuously dried at 60 °C for 1 h.

### 2.4. MFC Construction and Operation

**Figure 1:**
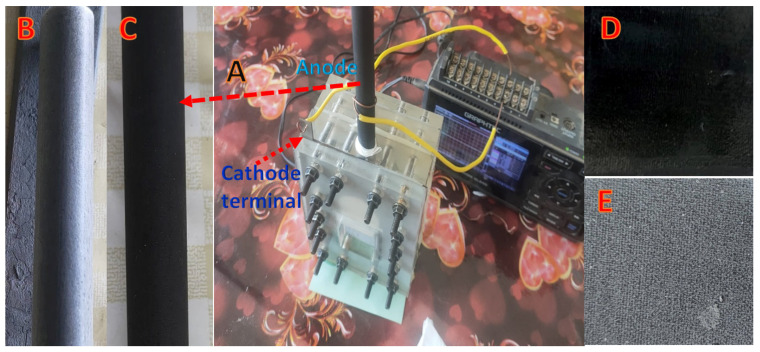
Photo images of the used cell; (**A**), the graphite rod after covering by PAN electrospun nanofibers; (**B**) and after calcination; (**C**), the bared active surface of the used cathode; (**D**), and the Pt-free cathode surface after covering by PVDF electrospun nanofiber layer; (**E**).

The use of air cathode qualified the MFC to be a cost effective and a portable device. The PVDF nanofibers-covered carbon cloth Pt-loaded cathode was used; the surface covered by the electrospun nanofiber layer was faced to the air side to control the oxygen diffusion and prevent water leakage, while the other side of which was loaded by Pt/C particles (0.5 mg/cm^2^) was facing the water side. The prepared anodes were immersed in 80 mL analoyte solution (sugarcane wastewater) after purging by nitrogen for 10 min. Figure 1 illustrates the used cell and other components. High corrosion resistance stainless steel sheet was used as a current collector at the cathode side, while the graphite rod was connected directly to the data logger. For comparison, 3.5 × 3.5 cm carbon cloth and carbon papers were utilized as anodes at similar conditions. All the experiments were conducted at ambient temperature. In order to immobilize the microorganisms on the anode for a certain time until the open circuit voltage (OCV) was stabilized and the two half reactions equilibrium was achieved, the media was injected into an anaerobic anode chamber. The cell was then operated by connecting the anode and cathode collectors with the potentiostate. The anode and cathode potentials were measured using an Ag/AgCl reference electrode. The potentiostate, which linked the working electrode to the cathode and the counter electrode and reference electrodes to the anode, was used to measure the OCV; the data were recorded by a GL220 midi-logger. By adjusting the circuit’s external resistance (load), utilizing potentiostate liner sweep voltammetry, the cell circuit was closed once OCV was reached to the stabilization in order to describe the current as a function of voltage (LSV). At a scan rate of 1 mVs^−1^, the current–voltage relationship (I-V) was established starting from the observed highest OCV to a zero voltage.

### 2.5. Characterization

The scanning electron microscope (SEM Hitachi S-7400, Hitachi Ltd, Hitachi, Japan) in the Central lab for Microanalysis and Nanotechnology, Minia University was used to characterize the surface morphology of the anode material after finishing the experiment. Briefly, the anode material was immersed in hot water for one night to remove any substrates deposited on the surface. Later on, after vacuum drying at 50 °C for two hours, a small piece was cut carefully from the rod surface and subjected to the analysis. To improve the imaging of samples, the samples were coated by a very thin layer of gold; deposition time was 10 s. Creating a conductive layer of metal on the sample inhibits charging, reduces thermal damage, and improves the secondary electron signal required for topographic examination in the SEM. Wastewater characterization was conducted in the central labs of Sanitation and Drinking Water Company in Minya city, Egypt. Cyclic voltammetry and linear scan measurements performed using VersaStat 4 instrument (AMETEK Scientific Instrument, Princeton Applied Research, Berwyn, PA, USA). In these measurements, the cathode was assigned to be a working electrode while both of counter and reference electrode cables were connected to the anode. The polarization curves were conducted by performing linear scan starting from the open cell voltage to zero volt at a scan rate of 0.001 V/s at room temperature. The cyclic voltammetry measurements, in the presence of the wastewater, were performed within a potential window of −1–1 V. Water contact angle measurement was performed using a contact angle meter (Biolin Scientific, Theta Flex, Gothenburg, Sweden). The measurement was conducted on the deposited PVDF electrospun nanofiber layer, attaching the utilized cathode without any further pretreatment. The measurements were performed 10 times. Each time, the left and right angles were measured and the mean values have been calculated.

## 3. Results

### 3.1. Hydrophobic Cathode Development

**Figure 2:**
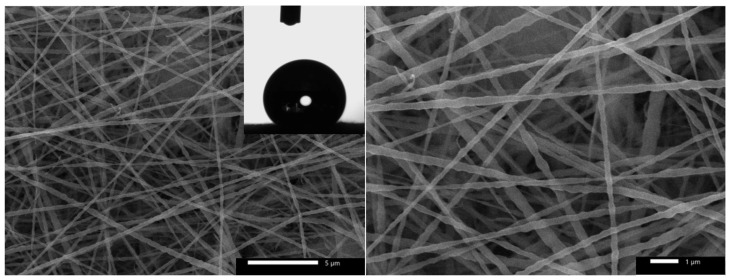
Two SEM images of the electrospun PVDF layer on the carbon cloth cathode, the inset displays water contact angle measurement of the covered cathode surface.

Beside the main function (ions exchange), in the conventional fuel cells devices, cation exchange membrane is utilized for other purposes. First, it prevents oxygen (or other electron acceptor compounds) from reaching and solvation in the anode solution which decreases losses of the used fuel by direct oxidation far from the anode surface. Second, in the air-cathode cells, the membrane prevents leakage of the fuel through the cathode layer. However, membrane utilization strongly increases the cell internal resistance which negatively affects the generated power from the cell. In industrial wastewater-driven microbial fuel cells, the fuel is dissolved organic pollutants, so oxygen solvation in the anode chamber due to pushing through the cathode layer is an unimportant factor to be considered, and especially avoiding using the membrane sharply improves the cell performance. Therefore, if water leakage, through the cathode, could be controlled in a membrane-less MFC, the internal resistance will be distinctly diminished which will greatly improve the generated power. PVDF is a well-known hydrophobic polymer [39]. Moreover, oxygen molecules can easily pass through a thin electrospun nanofiber layer due to the high porosity [40]. Based on these facts, the used cathode in this study has been modified. Typically, a thin layer (~5 min electrospinning time) of PVDF nanofibers was deposited on the Pt-free surface of the used carbon cloth cathode; panel E in Figure 1. Figure 2 displays two magnifications SEM images of the PVDF nanofibers layer. As shown, smooth and beads-free PVDF nanofibers were formed. Moreover, the average diameter of the deposited nanofibers was determined to be 210 ± 15 nm which reflects the high porosity of the attached layer. Interestingly, as shown in the inset, the attached polymeric layer is highly hydrophobic. Numerically, the measured water contact angle was 131°. Consequently, it can be claimed that, although the deposited PVDF nanofiber layer can allow oxygen molecules to pass easily due to the high porosity, water molecules cannot penetrate through this layer because of the high hydrophobicity. Therefore, exploiting the modified cathode in a membrane-less MFC will not be associated with the water leakage which was observed experimentally. The porosity of the deposited layer was estimated using imageJ software; the results indicated that the PVDF electrospun nanofiber layer has an average porosity of ~69%.

### 3.2. Performance of the Assembled MFCs

**Figure 3:**
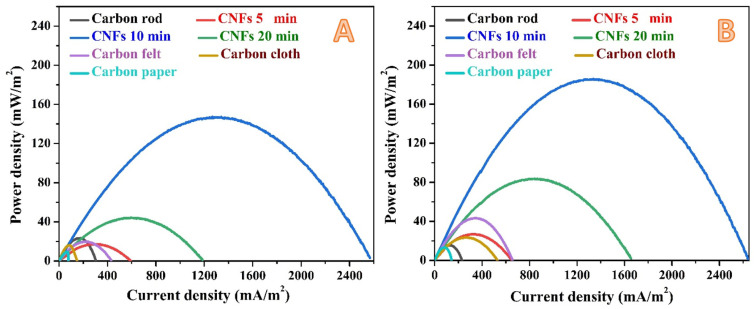
Generated power density using the proposed CNFs-covered graphite rod with different electrospinning times after 2 h; (**A**) and 1 day; (**B**) working time. Carbon cloth, carbon felt and carbon paper have been utilized for comparison.

Figure 3 represents the polarization curves for the assembled microbial fuel cells using bare and carbon nanofibers-covered (5, 10, and 20 min electrospinning time) graphite rods. Moreover, commercial carbon paper, carbon cloth and carbon felt were used as anodes to properly evaluate the performance of the proposed anodes. The results were also summarized in Table 2. The power and current densities were normalized to the cathode surface area. Measurements were carried out after 2 h (Figure 3A) and one day (Figure 3B) working time. As shown, the commercial anode materials show relatively small current and power densities. As documented in Table 2 that, after 2 h operation time, a maximum power density of 9 ± 0.3, 16 ± 0.3 and 19 ± 0.7 mW/m^2^ was generated from MFCs assembled using anode from carbon paper, carbon cloth, and carbon felt, respectively. For the same sequence, the detected maximum current densities were 77 ± 3, 149 ± 5 and 431 ± 9 mA/m^2^, respectively. Considering the short cells running time, the generated power and current densities are very satisfactory compared to the literature. Moreover, within this short working time, the results affirm the priority of carbon felt over the other two anodes which was also reported in the literature [41].

On the other hand, the naked graphite rod reveals competitive results compared to the commercial anodes during this short time period. As shown, the detected power and current densities from the graphite rod-based cell were 23 ± 0.4 mW/m^2^ and 303 ± 7 mA/m^2^, respectively. Considering the low cost of the graphite rod compared to the commercial used anodes, these results discover a new, cheap, easily handled and effective anode for the MFCs if a proper surface modification could be achieved. The deposition of thin layer of carbon nanofibers (5-min electrospinning time) reveals distinct enhancement especially in the form of current density (591 ± 9 mA/m^2^) compared to the commercial anodes and unclad graphite rod. Interestingly, increasing the PAN electrospinning time to 10 min resulted in a wide jump in both of power and current densities. As shown in Figure 3A and Table 2, the determined power and current densities were 147 ± 4.5 mW/m^2^ and 2565 ± 24 mA/m^2^, respectively which represent around eight and six folds of increasing power and current densities compared to the best commercial anode (carbon felt) at this working time, respectively. However, increasing the electrospinning time to 20 min showed a negative impact on the cell performance. The MFC assembled using a graphite rod covered by a 20-min electrospun PAN nanofiber could generate power and current densities of 44 ± 1.5 mW/m^2^ and 1185 ± 9 mA/m^2^, respectively. Although this anode could almost double the generated energy compared to the best commercial anode, its performance is relatively low compared to the 10-min anode.

An almost similar performance trend was observed after 1 day working time, as can be seen in Figure 3B and Table 2. However, there are some considerable observations to be made:

- Despite little improvement in the generated power and current densities upon increasing the working time to one day, carbon paper still has the least anode performance.
- The graphite rode sheathed by carbon nanofiber layer at electrospinning time of 10 min still keeps its distinction by generating power and current densities of 185 ± 7.4 mW/m^2^ and 2640 ± 26 mA/m^2^, respectively. These results represent around 25% increase, in term of power, compared to 2 h working time, and 800% compared to the best commercial anode (carbon felt) at the same working time (1 day). However, upon working time increase, the increase in the current density does not match the power density increasing fashion; a little increase in the current density was observed compared to 2 h working time. This finding can be attribute to mass transfer limitation of reaching the organic molecules to the attached microorganisms amongst the carbon nanofiber layers. In other words, increasing the time led to increase the number of the microorganisms in the biofilm, however, as the current represents the liberated electrons due to organic compounds metabolism at the anode surface, the slight change in the current density is attributed to a mass transfer limitation process.
- Among all investigated anodes, only the naked graphite rod showed lower performance upon time increase; the power and current densities decreased from 23 ± 0.4 mW/m^2^ and 303 ± 7 mA/m^2^ (at 2 h) to 15 ± 0.7 mW/m^2^ and 228 ± 6 mA/m^2^ (at 1 day), respectively. This finding might reflect low adhesion force between the attached microorganisms and the anode surface which results in a high releasing rate.
- There is an observable enhancement in the generated power from the 5-min and 20-min anodes. However, the performance of the later was almost doubled due to increasing the working time to 1 day which represents more attaching of the microorganisms at this anode compared the thin carbon nanofiber layer one (5-min).

### 3.3. Used Anodes Morphology

**Figure 4:**
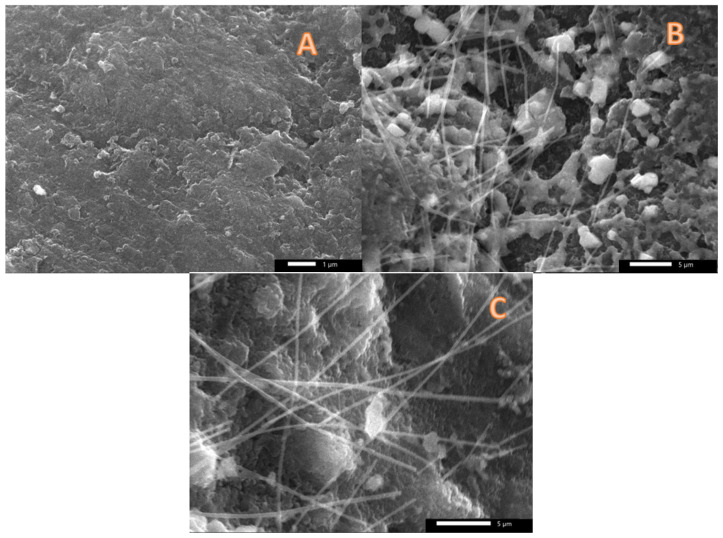
SEM image of the used CNFs-covered graphite rode anodes prepared at 5 min; (**A**), 10 min; (**B**) and 20 min; (**C**).

SEM images for the used graphite anodes (5, 10, and 20 min electrospinning time) after 1-day cell working time are represented in Figure 4. The images supported the polarization curve results (Figure 3) as well as the aforementioned explanations as follow: As shown in Figure 4A, for the 5-min anode, we could not find attached cells or nanofibers. Considering the high shrinkage in the nanofiber size taking place during the graphitization process due to polymer decomposition and releasing of many compounds, at this short electrospinning time, it expected that the formed carbon nanofibers will be in the form of small discrete spots on the surface of the graphite rod. Therefore, this finding supports the aforementioned claim about the poor microorganisms’ adhesion with the surface of the bare graphite rode. Meanwhile, the image can explain the low performance of this graphite rod anode.

Figure 4B,C represent SEM images of the 10- and 20-min anodes, respectively. As shown, in the image representing the anode giving the best performance (10-min anode; Figure 4B), carbon nanofibers could be seen with numerous microorganisms. On the other hand, as shown in Figure 4C which demonstrates the SEM image of the 20-min anode, the detected number of microorganisms is very small compared to the optimum anode.

Accordingly, based on the results in Figure 3 and Figure 4, it can be claimed that at the optimum electrospinning time (10 min), the deposited carbon nanofiber layer possesses a convenient pore size allowing the microorganisms to be inserted amongst the nanofiber layer. Therefore, a highly conductive 3D network is created to transfer the exerted electrons from the microorganisms that has been reflected into a high enhancement in the generated power and current densities. While, at a 20-min electrospinning time, the high packing density of the produced carbon nanofiber layer resulted in decreasing the pore size to be small to allow passing many microorganisms.

### 3.4. Cell Electrical Characteristics

**Figure 5:**
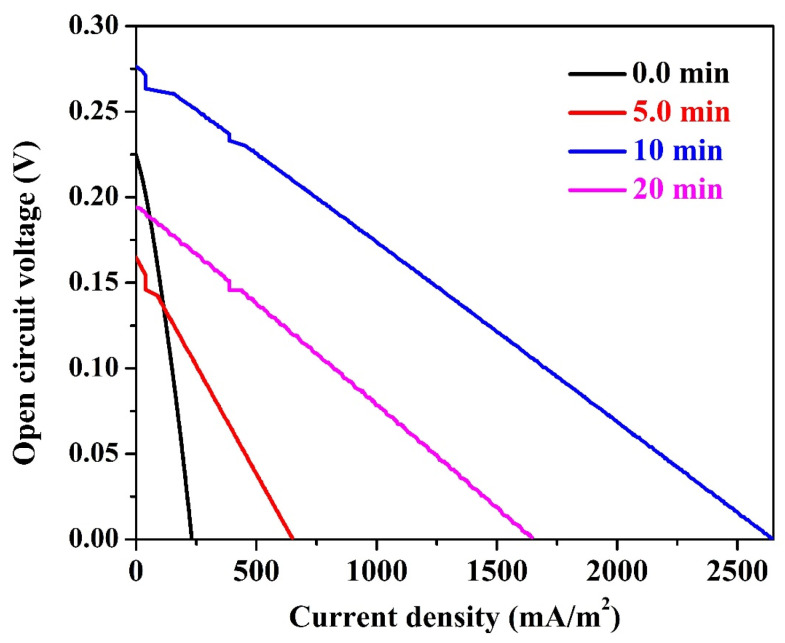
Relationship between OCV and current density for MFC equipped with naked and sheathed graphite rods (at different electrospinning times) anodes.

The most important two electrical characteristics of the MFC are the cell internal resistance and the anode surface electrical properties. The internal resistance of the MFC refers to both the resistance of electrons to flow through the electrodes and interconnections as well as the resistance of ions to flow from cathode to anode through the cation exchange membrane (if exists) and anodic electrolytes [42,43]. The linear polarization curve may be used to calculate the internal resistance (*R*_int_) of the MFC, which equals the slope of the curve (*E/I*) as illustrated in Figure 5. Overall, the internal resistance in the conventional MFCs can be divided into three regions through polarization curve. The first zone is called “activation zone” which appears at low current and high potential part in the polarization curve. Within this region, the generated electrons from the exo-electrogenic microorganisms need to overcome the back potential added to the cell from the external power supply. Second zone called “Ohmic resistance zone”, which has a vital role in determining the point of the maximum achievable power. Third zone, called mass transfer resistance zone, where, a very high drop voltage until zero value of potential, at the maximum current [44]. Very interestingly, the obtained linear scan voltammetry curves for the assembled cells behave as Ohmic resistance with trivial resistance values as shown in the figure. Numerically, the determined cell resistances are 1.88, 0.495, 0.202 and 0.229 mΩ for the MFCs having graphite rods anodes covered by carbon nanofibers produced at electrospinning times of 0, 5, 10 and 20 min, respectively. Obtaining just Ohmic resistances with trivial values can be attributed to two main reasons. First, the success in compiling membrane-less cells due to the newly added characteristic for the utilized cathodes; hydrophobic nature. Second, the excellent conductivity of the used graphite rods as well as the formed 3D biofilm layer as a result of the known very high conductivity of the carbon nanofibers.

**Figure 6:**
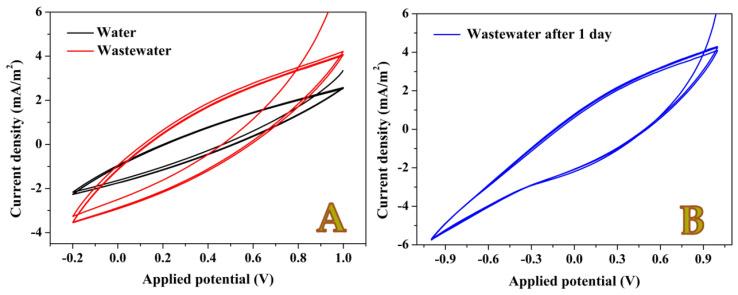
Cyclic voltammetry measurements for CNFs 10 min anode–based MFC using distilled water and wastewater as anolytes after 1 h assembling time; (**A**), and in case of wastewater after 1 day working time; (**B**). Scan rate 50 mV/s at 25 °C.

The electrical properties of the anode surface can be investigated by cyclic voltammetry measurements. Figure 6 displays the cyclic voltammograms of the 10-min graphite rod MFC in presence of organic pollutants-free water and the utilized wastewaters. Moreover, the measurements have been conducted after short and long (1 day) operating times; Figure 6A,B. As shown in the obtained cycles, no redox peaks could be obtained even through large potential window (−1 to 1 V; Figure 6B). This finding concludes that the surface of the proposed anodes is free from any oxidizable compounds (e.g., metallic compounds). Therefore, it can be confidently claim that the observed current in the used cells is generated from the biofilm layer. Indeed, knowing the aerobic and anaerobic microorganisms in the utilized wastewater can help in improvement the cell performance. Therefore, this will be the future work.

## 4. Conclusions

Commercial graphite rods can be exploited as highly effective and cheap anodes in the microbial fuel cells after decoration by a thickness-optimized carbon nanofiber layer. Deposition of carbon nanofiber layer can be performed by electrospinning process using the most widely used carbon nanofiber precursor; polyacrylonitrile (PAN)/N,N-Dimethylformamide (DMF) solution. However, to get the best performance, the electrospinning time should be adjusted; 10 min with the used graphite rod size reveals the optimum value. Membrane-less microbial fuel cell can be compiled using hydrophobic cathode coated by poly(vinylidene fluoride) electrospun nanofiber layer. The proposed anodes are highly recommended to replace the conventional commercial anode materials (e.g., carbon paper, carbon cloth and carbo felt) to generate electricity from organic pollutants-containing wastewaters with simultaneous treatment in the coming promised energy device; microbial fuel cells.

## Figures and Tables

**Table 1:** Chemical Characterizations of Wastewater.

pH	COD (mg/L)	Na^+^ (mg/L)	Ca^+2^ (mg/L)	Total P (mg/L)	SO_4_^−2^ (mg/L)	K^+^ (mg/L)	TDS (mg/L)	Conductivity (µS/cm)
6.5 ± 0.5	4000 ± 70	360 ± 25	33 ± 2	18 ± 1.5	28 ± 2.5	350 ± 15	1760 ± 55	3600 ± 95

**Table 2:** Summary of the generated power density using different anode materials after 2 h and 1 day working times.

	Carbon Paper		Carbon Cloth		Carbon Felt		Graphite Rod		CNFs 5 min		CNFs 10 min		CNFs 20 min	
	2 h	1 day	2 h	1 day	2 h	1 day	2 h	1 day	2 h	1 day	2 h	1 day	2 h	1 day
**P.D** **(mW/m^2^)**	9 ± 0.3	13 ± 0.3	16 ± 0.3	43 ± 1.4	19 ± 0.7	23 ± 0.7	23 ± 0.4	15 ± 0.7	17 ± 0.1	27 ± 0.9	147 ± 4.5	185 ± 7.4	44 ± 1.5	83 ± 4.3
**C.D** **(mA/m^2^)**	77 ± 3	141 ± 7	149 ± 5	525 ± 10	431 ± 9	650 ± 11	303 ± 7	228 ± 6	591 ± 9	645 ± 10	2565 ± 24	2640 ± 26	1185 ± 9	1650 ± 19

## Data Availability

Not applicable.
